# StAR Protein Deficiency in Clinical Practice: A Case Series From Saudi Arabia

**DOI:** 10.1155/crie/8489134

**Published:** 2026-01-06

**Authors:** Abeer Alabduljabbar, Dania Farooq, Sara Abid, Sara Aljazaeri, Raghad Alhuthil, Afaf Alsagheir

**Affiliations:** ^1^ Department of Pediatrics, King Faisal Specialist Hospital and Research Centre, Riyadh, Saudi Arabia, kfshrc.edu.sa; ^2^ College of Medicine, Alfaisal University, Riyadh, Saudi Arabia, alfaisal.edu

**Keywords:** 46XY disorders of sex development, cholestasis, congenital adrenal hyperplasia, consanguinity, StAR deficiency, steroidogenesis

## Abstract

**Objectives:**

Steroidogenic acute regulatory (StAR) protein deficiency is a rare autosomal recessive disorder that disrupts steroid hormone biosynthesis, resulting in congenital adrenal hyperplasia (CAH) and variations in sexual development. However, limited data is available in Saudi Arabia. Therefore, this study describes the clinical and genetic findings of seven Saudi patients with StAR deficiency.

**Methods:**

This case series was conducted at King Faisal Specialist Hospital and Research Centre (KFSHRC) in Riyadh, Saudi Arabia.

**Results:**

All seven patients were born to consanguineous parents, most commonly first cousins. Five patients had a 46,XY karyotype, and two had a 46,XX karyotype. All were clinically diagnosed with CAH due to StAR deficiency. Despite their chromosomal sex, all presented with a female external phenotype. Clinical features ranged from typical female genitalia to varying degrees of feminization with bilateral inguinal gonads or undescended testes in 46,XY individuals. Most patients exhibited electrolyte disturbances and chronic salt‐wasting. Interestingly, two cases presented with neonatal cholestatic jaundice. Genetic testing confirmed homozygous pathogenic or likely pathogenic *STAR* variants in all cases. Dysmorphism occurred in one patient with (c.402T >G, p.Tyr134Ter) mutation. The five 46,XY patients underwent bilateral gonadectomy. All patients remain clinically stable on long‐term steroid replacement.

**Conclusion:**

This study highlights the diverse clinical spectrum of StAR deficiency, ranging from early adrenal crisis to DSD and atypical presentations such as cholestasis. The findings underscore the importance of early genetic diagnosis and counseling, particularly in consanguineous populations, and point to the need for continued research into the clinical and molecular complexity of StAR deficiency.

## 1. Introduction

Steroid hormones are essential for maintaining homeostasis, supporting reproductive development, and regulating numerous physiological processes. Despite their varied functions, all steroid hormones originate from a common precursor, cholesterol. The steroidogenic acute regulatory (StAR) protein plays a pivotal role in steroidogenesis by facilitating the transport of cholesterol into the mitochondria, where it is converted to pregnenolone, the rate‐limiting step in steroid hormone biosynthesis [1].

Mutations in the *STAR* gene lead to a dysfunctional StAR protein, impairing cholesterol transport and disrupting steroid hormone production [2]. Clinically, this condition, known as StAR deficiency, presents as primary adrenal insufficiency and disorders of sex development (DSDs). Individuals with a 46,XY karyotype may present with a phenotypically female appearance despite having a genetically male profile [3].

Diagnosing StAR deficiency can be challenging due to its clinical overlap with other forms of congenital adrenal hyperplasia (CAH), particularly 21‐hydroxylase deficiency, the most common form of CAH [4]. While biochemical markers such as low cortisol and aldosterone levels, along with elevated ACTH and plasma renin activity, may suggest adrenal insufficiency, definitive diagnosis requires genetic confirmation through identification of mutations in the *STAR* gene [5].

Despite its clinical significance, StAR deficiency remains underreported, particularly in Saudi Arabia, with limited studies available [6–8]. The high rate of consanguinity in countries like Saudi Arabia increases the risk of autosomal recessive disorders such as this [9]. However, limited data exist on the clinical spectrum, genetic variants, and long‐term outcomes of *STAR*‐related adrenal insufficiency in the region.

This study aims to address this knowledge gap by presenting a case series of patients with genetically confirmed *STAR* mutations followed at a large referral center in Saudi Arabia. Through a detailed clinical and genetic analysis, the study underscores the importance of early diagnosis, genetic counseling, and the need for increased awareness to improve outcomes and inform future research in populations with a high prevalence of consanguinity.

## 2. Patients and Methods

We retrospectively reviewed seven patients with genetically confirmed StAR protein deficiency, all of whom were comprehensively evaluated at King Faisal Specialist Hospital and Research Centre (KFSHRC) in Riyadh, Saudi Arabia. All patients were born to consanguineous parents, primarily first cousins. The series consisted of five patients with a 46,XY karyotype and two with a 46,XX karyotype (Table 1).

**Table 1:** Comparative clinical features of patients with STAR gene mutation.

Case	Gender assignment	Phenotype	Ambiguous genitalia	Karyotype	Consanguinity	History of adrenal insufficiency	Hormonal levels	Imaging findings	Management/outcome
1	Female	Jaundice, hyperpigmentation, no palpable gonads	Yes *phenotypically female*l	46,XY	Yes	Positive	Low cortisol, high ACTH	Abdominal ultrasound: bilateral inguinal gonads, hydrocele	Hydrocortisone, fludrocortisone; improved with no concerns Bilateral gonadectomy.
2	Female	Jaundice, dehydration, hyperpigmentation	No	46,XX	Yes	Positive	Low cortisol, high ACTH, normal 17‐OHP	Ultrasound: normal uterus, no right ovary visualized	With hydrocortisone, fludrocortisone, doing well.
3	Female	Dysmorphic features, hyperpigmentation	Yes *phenotypically female*	46,XY	Yes	Positive	Low cortisol, high ACTH, low aldosterone, low 17‐OHP	Ultrasound: inguinal testes, no uterus or female gonads	Hydrocortisone, fludrocortisone; improved Bilateral gonadectomy.
4	Female	Hyperpigmentation, normal female phenotype	Yes *phenotypically female*	46,XY	Yes	Positive	Normal ACTH, normal renal profile	Ultrasound: inguinal testes, no uterus or female gonads	Stable on prednisone, fludrocortisone; doing well. Bilateral Gonadectomy.
5	Female	Female phenotype with right testis palpable	Yes *phenotypically female*	46,XY	Yes	Positive	High ACTH, low cortisol, low 17‐OHP	MRI: undescended left testis, no internal female organs	Stable on prednisolone + fludrocortisone. Bilateral orchidectomy severe obesity, planning for semaglutide and possible surgery
6	Female	Normal female phenotype, no palpable gonads	No	46,XX	Yes	Positive	High ACTH, low cortisol	Ultrasound: normal internal female organs	Hydrocortisone, fludrocortisone; doing well
7	Female	Female phenotype, no palpable gonads	Yes *phenotypically female*	46,XY	Yes	Positive	Low cortisol, low aldosterone, high ACTH	Ultrasound: inguinal testes, no uterus or female gonads	Hydrocortisone, fludrocortisone; doing well Bilateral gonadectomy.

Each patient underwent a detailed clinical, hormonal, radiological assessment and chromosomal analysis (karyotyping) was performed in all cases during their routine visits to confirm genetic sex and guide further management. Hormonal profiling included ACTH, cortisol, aldosterone, 17‐hydroxyprogesterone, and additional relevant steroid precursors. Radiological imaging, such as ultrasound and MRI was used to evaluate the presence and morphology of internal reproductive structures and gonads.

Genetic testing was conducted using Whole Exome Sequencing (WES) to identify mutations in the *STAR* gene following karyotype evaluation. WES was selected as the first‐line molecular test because targeted gene panels are not routinely available, and to provide comprehensive coverage of the heterogeneous genetic etiologies encountered in our cohort, and to avoid delays associated with sequential targeted testing.

Variants classification was reported as per the ACMG “American College of Medical Genetics” classifications [10].

## 3. Case Presentation

### 3.1. Case 1

A phenotypic female neonate (birth weight 2.4 kg) developed cyanosis and bradycardia at 10 min of life, requiring intubation and NICU admission. She had generalized jaundice, hyperpigmentation, normal female external genitalia, and recurrent hypoglycemia. Persistent cholestasis required ursodeoxycholic acid. Critical samples showed markedly low cortisol (37–42 nmol/L), leading to a diagnosis of primary adrenal insufficiency and initiation of hydrocortisone. At 50 days of life, repeat labs showed ACTH 1645 ng/L, low cortisol, mild hyponatremia, hyperkalemia, and elevated liver enzymes. Karyotyping revealed 46,XY. Ultrasound showed bilateral inguinal testes with no Müllerian structures. WES confirmed homozygous *STAR* mutation c.544C >T (p.Arg182Cys). After adjusting steroid replacement, cholestasis and electrolyte disturbances resolved. She is now 2 years old and clinically stable.

### 3.2. Case 2

A full‐term female (birth weight 3 kg) developed jaundice, pale stools, vomiting, and hyperpigmentation at 1 month. Labs showed hyponatremia, hyperkalemia, hypoglycemia, direct hyperbilirubinemia, and metabolic acidosis. Endocrine workup revealed ACTH 1200 ng/L, very low cortisol, and 17‐OHP< 0.1 nmol/L. Karyotype was 46,XX. Ultrasound visualized a uterus and left ovary. She was diagnosed with CAH with salt‐wasting features and started on hydrocortisone and fludrocortisone. WES confirmed homozygous *STAR* mutation c.402T >G (p.Tyr134Ter). A maternal cousin had the same condition. The patient was raised female and underwent bilateral laparoscopic orchidectomy.

### 3.3. Case 3

A girl born at 36 weeks developed recurrent neonatal hypoglycemia and was re‐admitted for poor feeding. She had dysmorphic features, generalized hyperpigmentation, and subsequently developed hyponatremia and hyperkalemia. Endocrine evaluation showed ACTH > 400 ng/L, very low cortisol, low aldosterone, and 17‐OHP < 0.1 nmol/L. Karyotype revealed 46,XY, confirmed by FISH. HCG stimulation showed no testosterone response. Ultrasound demonstrated bilateral inguinal gonads. WES identified homozygous *STAR* mutation c.402T>G (p.Tyr134Ter). She underwent bilateral orchidectomy; pathology confirmed prepubertal testes. She remains clinically stable on replacement therapy at age 10.

### 3.4. Case 4

A woman diagnosed with adrenal insufficiency at 10 days old had hyponatremia, hyperkalemia, elevated ACTH, and low cortisol, and was started on hydrocortisone and fludrocortisone. At age 10, karyotype revealed 46,XY, and ultrasound showed inguinal gonads. She was raised female and underwent bilateral gonadectomy. WES later confirmed a homozygous likely pathogenic *STAR* mutation c.790C >T (p.Gln264Ter), consistent with lipoid CAH. She remains stable on lifelong steroid therapy.

### 3.5. Case 5

A 14‐year‐old female‐raised patient was referred at 2 weeks for suspected DSD. Although external genitalia appeared female, karyotype was 46,XY. The right testis was palpable; the left testis was identified in the inguinal canal on imaging. Hormonal evaluation showed very high ACTH (2000 ng/L), low cortisol, and low 17‐OHP. MRI and ultrasound confirmed bilateral testes. WES revealed homozygous *STAR* mutation c.545G > A (p.Arg182His). Bilateral orchidectomy confirmed prepubertal testes. She developed severe obesity (BMI 51) and will start semaglutide, with bariatric surgery under consideration. She remains on prednisolone and fludrocortisone.

### 3.6. Case 6

A 17‐year‐old girl with CAH diagnosed at birth was referred for follow‐up. She had primary adrenal insufficiency with prior electrolyte imbalance and was maintained on hydrocortisone 5 mg three times daily (11.8 mg/m^2^/day) and fludrocortisone 0.1 mg daily. She had a normal female phenotype with no palpable gonads. Laboratory results showed markedly elevated ACTH (501 ng/L; normal 5–60 ng/L) and undetectable cortisol (<1.5 nmol/L). Karyotype was 46,XX, and abdominal ultrasound demonstrated normal internal female reproductive organs. WES revealed a homozygous pathogenic STAR mutation (c.545G > A, p.R182H) (Table 2), confirming congenital lipoid adrenal hyperplasia (CLAH).

**Table 2:** Genetic variants identified in patients with positive *STAR* gene (NM_000349.3).

Case	Karyotype	Variant	Exon	ACMG class	Coding impact	dbSNP ID	Zygosity
1	46,XY	c.544C >T, p.Arg182Cys	5	P	missense	rs369232492	Homozygous
2	46,XX	c.402T >G, p.Tyr134Ter	4	P	nonsense	rs144881901	Homozygous
3	46,XY	c.402T >G, p.Tyr134Ter	4	P	nonsense	rs144881901	Homozygous
4	46,XY	c.790C >T; p.Gln264Ter	7	LP	nonsense	NA	Homozygous
5	46,XY	c.545G >A; p.Arg182His	5	P	missense	rs104894086	Homozygous
6	46,XX	c.545G >A; p.Arg182His	5	P	missense	rs104894086	Homozygous
7	46,XY	c.545G >A; p.Arg182His	5	P	missense	rs104894086	Homozygous

*Note*: ACMG Class, American College of Medical Genetics and Genomics classification; dbSNP, single nucleotide polymorphism database identifier.

Abbreviations: LP, likely pathogenic; NA, not available; P, pathogenic.

### 3.7. Case 7

A 23‐year‐old woman, diagnosed with CAH in infancy after presenting with hyponatremia and hyperkalemia, was referred for evaluation. Earlier records noted low cortisol, elevated renin and ACTH, subnormal aldosterone, and 17‐hydroxyprogesterone > 0.4 nmol/L (normal > 1 nmol/L), supporting primary adrenal insufficiency. She is maintained on hydrocortisone 7.5 mg twice daily and fludrocortisone 0.1 mg daily. She had a normal female phenotype on examination with no palpable gonads. Karyotyping revealed 46,XY. Pelvic ultrasound showed absent uterus and internal female structures, with bilateral inguinal masses suggestive of undescended testes. WES identified the same homozygous *STAR* mutation (c.545G > A; p.R182H) (Table 2), confirming CLAH.

## 4. Discussion

In our case series, all patients had homozygous pathogenic or likely pathogenic variants in *STAR*, consistent with autosomal recessive inheritance. Most patients presented during the neonatal period with features of adrenal insufficiency, hyponatremia, hyperkalemia, hypoglycemia, and elevated ACTH, leading to a diagnosis of CLAH. These findings align with previous reports of classic *STAR* deficiency, where early‐onset adrenal insufficiency is the hallmark, often accompanied by skin hyperpigmentation, vomiting, diarrhea, failure to thrive, and severe electrolyte disturbances [2, 11].

An important and consistent clinical feature observed in our series was generalized hyperpigmentation, attributed to elevated ACTH and melanocyte‐stimulating hormone (MSH). This sign, particularly when observed in neonates or infants, should prompt urgent endocrine evaluation, including measurements of cortisol, aldosterone, ACTH, and plasma renin activity. Patients in our series showed a hormonal profile of low cortisol and aldosterone, markedly elevated ACTH, and, when tested, low or undetectable 17‐hydroxyprogesterone, consistent with the diagnosis of CLAH [6].

All 46,XY individuals in our series exhibited female external genitalia, underscoring the severe disruption of testicular steroidogenesis due to *STAR* mutations. The failure to produce testosterone during fetal life results in complete sex reversal despite the presence of XY chromosomes. This is driven by cholesterol accumulation in steroidogenic tissues, especially the adrenal cortex and fetal testes, causing cellular damage and loss of function [5]. In contrast, 46,XX individuals typically appear phenotypically female at birth, as ovarian steroidogenesis does not initiate until puberty [3].

However, phenotypic variability has been documented. Altinkilic et al. [12] reported a 46,XY individual with adrenal insufficiency and a male phenotype, carrying compound heterozygous *STAR* mutations. This suggests that residual StAR activity, along with potential modifier genes or epigenetic factors, may influence phenotypic expression. Such findings emphasize the need for further research into genotype–phenotype correlations and the role of functional studies in atypical presentations.

The two‐hit model of CLAH offers a framework for understanding disease progression: Initially, limited steroidogenesis occurs via StAR‐independent pathways (first hit), followed by cytotoxic cholesterol accumulation that further impairs steroidogenesis (second hit). This model explains why 46,XY fetuses, whose Leydig cells are stimulated early by chorionic gonadotropin, are more severely affected than 46,XX individuals [3].

A noteworthy finding in this series was neonatal cholestasis in two patients (Cases 1 and 2), a rare but reported manifestation of *STAR* mutations [13, 14]. Case 1, phenotypically female, was later confirmed to have a 46,XY karyotype and intra‐abdominal testes, highlighting the importance of evaluating DSD in any female neonate with adrenal crisis and non‐palpable gonads. Similarly, Cases 3, 4, and 5, also 46,XY individuals raised as females, had ambiguous or female‐appearing genitalia, consistent with the known impact of *STAR* mutations on gonadal steroidogenesis [11].

Case 2, with a 46,XX karyotype and unambiguous female genitalia, presented with adrenal insufficiency and hyperpigmentation, illustrating how *STAR* mutations in 46,XX individuals may manifest solely with adrenal symptoms, making diagnosis more challenging [15]. In contrast, Cases 3 and 5 underscore the diagnostic complexity in patients with overlapping features of DSD and adrenal insufficiency. Such cases necessitate early endocrine and genetic evaluation to guide diagnosis, gender assignment, and management.

Regarding genetic findings, the mutation c.545G > A; p.Arg182His (Cases 5, 6, and 7) has been reported in several populations, including those from the Middle East [7, 8, 16–18], China [19], and international cohorts [20, 21], and may represent a regional founder variant. The c.790C > T; p.Gln264Ter variant (Case 4) was previously documented in a Saudi study [8], while c.544C > T; p.Arg182Cys (Case 1) has been reported across diverse populations, including the Middle East [7, 22], North Africa (Libya) [18], South Asia (India) [23], and East Asia [24, 25]. These patterns suggest the presence of recurrent mutational hotspots.

Although our sample size was small, several genotype–phenotype patterns were observed (Table 3). Both missense variants (p.Arg182His and p.Arg182Cys) and nonsense variants (p.Tyr134Ter and p.Gln264Ter) resulted in severe adrenal insufficiency and female external genitalia in 46, XY individuals, indicating near‐complete loss of StAR function. One patient with p.Tyr134Ter exhibited dysmorphic features, but a causal relationship to the variant cannot be assumed due to limited data. The repeated identification of p.Arg182His supports the possibility of a founder effect within the Saudi population.

**Table 3:** Phenotype–genotype patterns identified.

Variant	Type	Cases	Genotype → phenotype pattern
p.Arg182His (missense)	Severe LOF	5, 6, 7	46,XY → complete sex reversal; early adrenal insufficiency; no dysmorphism
p.Arg182Cys (missense)	Severe LOF	1	46,XY → complete sex reversal; cholestasis; adrenal crisis
p.Tyr134Ter (nonsense)	Null	2, 3	46,XY → complete sex reversal + dysmorphism; severe adrenal insufficiency
p.Gln264Ter (nonsense)	Null	4	46,XY → complete sex reversal; severe adrenal insufficiency

*Note*: LOF, loss‐of‐function mutations.

Several clinical observations also highlight gaps in diagnostic pathways. Case 4, diagnosed with adrenal insufficiency in infancy but not genetically confirmed until adulthood, illustrates a common diagnostic gap in older patients who lacked access to molecular testing. Similarly, Case 7 was initially diagnosed based on hormonal findings alone; later genetic testing revealed a 46,XY karyotype without Müllerian structures, consistent with *STAR*‐related CAH.

Gonadectomy was performed in several cases (2, 3, 4, and 5) after confirmation of 46,XY karyotype with intra‐abdominal or inguinal testes. Although the risk of malignancy in prepubertal undescended testes is low, prophylactic gonadectomy is often recommended in female‐reared patients due to potential germ cell tumor development in adulthood [26].

Despite adequate hormonal replacement, some patients developed complications. Notably, Case 5 developed morbid obesity (BMI 51), a known long‐term issue in CAH patients treated with chronic glucocorticoids [27]. This underscores the importance of multidisciplinary care, including endocrinology, nutrition, and, when appropriate, bariatric interventions.

Overall, this case series reinforces the clinical variability of *STAR*‐related CLAH, encompassing adrenal insufficiency, DSD, and less common features like neonatal cholestasis. Our findings highlight the importance of early molecular diagnosis, which facilitates individualized management, informed family counseling, and long‐term follow‐up.

Early recognition is crucial, as affected neonates are at high risk of life‐threatening adrenal crises due to salt‐wasting and electrolyte imbalances [6]. Prompt initiation of glucocorticoid and mineralocorticoid therapy can be lifesaving [28], and long‐term care should include routine monitoring for metabolic disturbances, growth issues, and fertility complications [29].

CLAH remains underrecognized, particularly in regions like Saudi Arabia, where consanguinity is common and increases the prevalence of autosomal recessive disorders [9]. Our findings support incorporating genetic testing, including karyotyping and WES, into the diagnostic workup of infants with adrenal insufficiency or ambiguous genitalia. Identifying *STAR* mutations provides critical guidance for both immediate treatment and future care planning.

## 5. Limitations

This case series is limited by its retrospective nature, small sample size, and single‐center design, which may limit the generalizability of the findings. Additionally, long‐term follow‐up data on patient outcomes, including pubertal progression, fertility potential, and psychosocial impact, were not uniformly available. Despite these limitations, the study contributes valuable insight into the clinical and genetic spectrum of StAR deficiency in a consanguineous population.

## 6. Conclusion

This case series illustrates the diverse clinical and genetic complexity of StAR deficiency in a Saudi series. The consistent presentation of female external genitalia in 46,XY individuals, alongside early‐onset adrenal insufficiency, emphasizes the essential role of StAR in steroidogenesis and sexual differentiation. The presence of neonatal cholestatic jaundice in two cases may also reflect distinctive regional or genetic patterns.

Given the high rate of consanguinity in the region, our findings underscore the need for early genetic diagnosis, targeted counseling, and lifelong endocrine management. Future research should focus on expanding cohort sizes, investigating long‐term outcomes, and exploring the influence of modifier genes and epigenetic factors. Incorporating next‐generation sequencing into standard diagnostic workflows may enhance diagnostic precision and patient care in StAR deficiency.

## Ethics Statement

This study was approved by the Research Ethics Committee at King Faisal Specialist Hospital and Research Centre (Decision No.: 2231110, Date: 11.04.2023).

## Consent

Written informed consent was taken for the genetic studies.

## Disclosure

The authors have nothing to report.

## Conflicts of Interest

The authors declare no conflicts of interest.

## Author Contributions

Surgical and medical practices: **Afaf Alsagheir**. Concept, design: **Abeer Alabduljabbar, Afaf Alsagheir**. Data collection or processing: **Dania Farooq, Sara Abid, Sara Aljazaeri**. Analysis or interpretation: **Raghad Alhuthil**. Writing: **Sara Abid, Sara Aljazaeri, Raghad Alhuthil, Dania Farooq, Abeer Alabduljabbar and Afaf Alsagheir**.

## Funding

No funding was received for this manuscript.

## Data Availability

Data can be available upon a reasonable request from the corresponding author.
